# Thermoelectric Power in Bilayer Graphene Device with Ionic Liquid Gating

**DOI:** 10.1038/srep20402

**Published:** 2016-02-08

**Authors:** Yung-Yu Chien, Hongtao Yuan, Chang-Ran Wang, Wei-Li Lee

**Affiliations:** 1 Institute of Physics, Academia Sinica, Nankang, Taipei 11529, Taiwan; 2 Geballe Laboratory for Advanced Materials, Stanford University, Stanford, California 94305, USA

## Abstract

The quest for materials showing large thermoelectric power has long been one of the important subjects in material science and technology. Such materials have great potential for thermoelectric cooling and also high figure of merit ZT thermoelectric applications. We have fabricated bilayer graphene devices with ionic-liquid gating in order to tune its band gap via application of a perpendicular electric field on a bilayer graphene. By keeping the Fermi level at charge neutral point during the cool-down, we found that the charge puddles effect can be greatly reduced and thus largely improve the transport properties at low *T* in graphene-based devices using ionic liquid gating. At (*V*_*ig*_, *V*_*bg*_) = (−1 V, +23 V), a band gap of about 36.6 ± 3 meV forms, and a nearly 40% enhancement of thermoelectric power at *T* = 120 K is clearly observed. Our works demonstrate the feasibility of band gap tuning in a bilayer graphene using ionic liquid gating. We also remark on the significant influence of the charge puddles effect in ionic-liquid-based devices.

Graphene-based device remains to be one of the most important subjects in both fundamental and applied researches. In fundamental sciences, many intriguing phenomena were discovered further extending the scope of our knowledge on material sciences, such as the quantum spin Hall phase in bilayer graphene^1^,^2^ and transverse magnetic focusing of electrons at room temperature in few layer graphene^3^. For practical applications, a number of experiments using graphene-based plasmonic and photonic devices draw many attentions recently^4^,^5^,^6^. Several optoelectronics devices have been proposed and demonstrated in graphene-based nanostructures, where fast electro-optics response and high tunability make it a wonderful platform for the application consideration. One important characteristic in a bilayer graphene (BLG) is the band gap tunability via a perpendicular electric field. A sizable band gap can be induced in a BLG by either chemical doping^7^ or dual-gated field effect^8^ to break the inversion symmetry of a BLG. It is, therefore, a challenging task to achieve an even bigger band-gap in graphene-based device, where higher on-off ratio and also thermoelectric power (TEP) can be largely enhanced^9^.

According to the Mott relation^10^, TEP of a material is proportional to the density of states (DOS) at Fermi level. In principle, if a giant band gap can be induced in a BLG, one expects to see a much larger DOS at the band edges leading to a huge TEP enhancement. In an earlier work, a four-fold enhancement in TEP at low temperature was demonstrated in a dual-gated BLG device using hexagonal boron nitride as a top gate dielectric material^9^. Unfortunately, the highest electric field can be applied on a BLG is only limited to about 1 V/nm mostly constrained by the breakdown voltage of the dielectric materials. One possible solution is to use high *κ* dielectric materials, such as HfO_2_. Nevertheless, deposition of oxide material on top of few layer graphene is known to greatly reduce the carrier mobility^11^. It is, therefore, an important subject to look for alternative approaches to fabricate top gate in graphene-based devices without degrading the graphene quality. In this report, we explored such a possibility by utilizing the ionic liquid (DEME-TFSI) gating technique in BLG devices. Ionic liquid is a molten salt at room temperature. Above its freezing point, the ions are mobile and can respond to an applied electric field. The ionic liquid side gate can serve as a top gate to introduce a large electric field on the top layer of BLG owing to the formation of the electric double layer (EDL)^12^,^13^. The thickness of EDL is typically around 1 nm, which provides a strong electric field gradient on the top surface of a material. The surface carrier density can be boosted up to the order of 10^14^ per cm^2^ that is difficult to achieve using regular oxide gating method. By controlling both the ionic liquid gate voltage and the bottom gate voltage, a sizable band-gap opening effect has been reported in a BLG device using ionic liquid gating^14^,^15^. Nevertheless, the associated TEP measurements remain lacking.

## Results and Discussions

**Figure 1:**
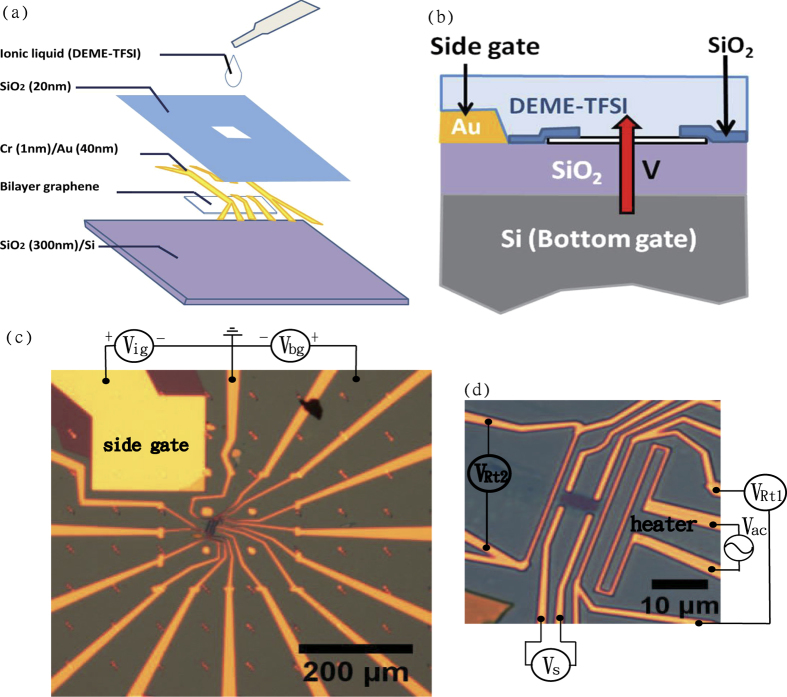
(**a**) An illustration of fabrication procedure for BLG device with ionic liquid gating. (**b**) A cross-sectional view of an ionic liquid gating BLG device. (**c**) An optical image at lower magnification. The large gold pad on the upper-left corner is the side gate for ionic liquid gating. (**d**) An optical image of a BLG device with ionic liquid gating. The gold contact pads are designed for both the TEP and charge transport measurements.

Figure 1(a) illustrates the fabrication procedure for a BLG device with ionic liquid gating. First, a microcrystal of BLG was mechanically exfoliated on a SiO_2_ (300 nm)/Si substrate followed by the gold contact pads fabrication using e-beam lithography. A thin layer of SiO_2_ (≈20 nm) were then evaporated on the BLG device to protect the device from the direct contact with the ionic liquid. Only a small window on the BLG was directly in contact with the ionic liquid. The cross-sectional view of the resulting device is shown in Fig. 1(b), where a side gate via the DEME-TFSI ionic liquid acts as a top gate. Both the side gate and bottom gate work simultaneously giving an electric field perpendicular to the BLG. Figure 1(d) shows an optical image of a BLG device we fabricated. The small rectangle at the center is the opening window to the BLG, where ionic liquid is directly in contact with the BLG. Other parts of the device were covered by a thin layer of SiO_2_ with thickness of about 20 nm. The contact pads of Ti/Au were fabricated by electron beam lithography, which comprises a local heater and two local thermometers (*V*_*t*1_, *V*_*t*2_) and 4 voltage leads on a BLG for electrical signal (*V*_*s*_) detection. The side gate and bottom gate are shown in Fig. 1(c) as *V*_*ig*_ and *V*_*bg*_, respectively. The large gold pad at the upper-left corner of the figure is the ionic liquid side-gate electrode. The ratio of the side-gate area to the BLG area should be maximized in order to achieve optimum ionic liquid gating effect. In our device shown in Fig. 1(c,d), the ratio of the side-gate area to the effective BLG area within the opening window is more than 10^3^, and thus the applied *V*_*ig*_ can be fully imposed upon the EDL at the surface of BLG.

**Figure 2:**
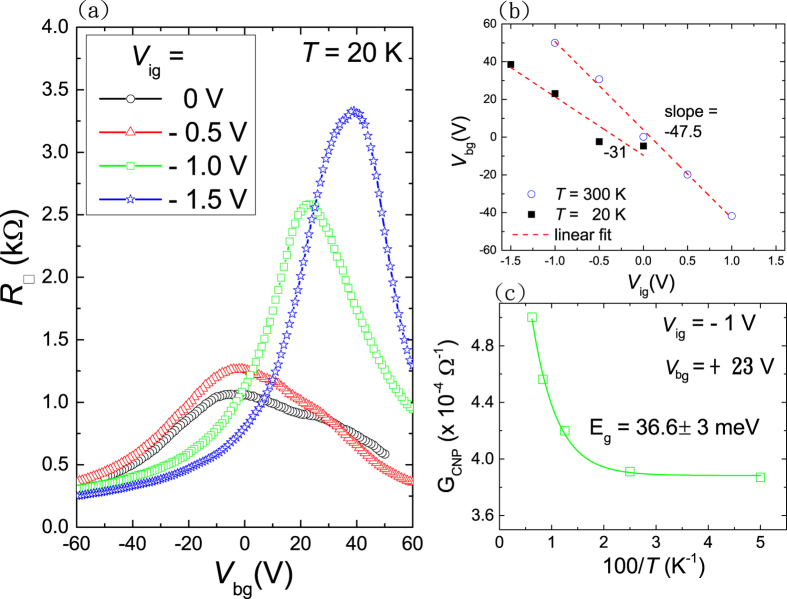
(**a**) Sheet resistance *R*_□_ versus bottom gate *V*_*bg*_ at *T* = 20 K under three different side gate voltages *V*_*ig*_. (**b**) The resistance peak location in (**a**) as a function of side gate voltage *V*_*ig*_ at *T* = 300 K and 20 K. (**c**) Conductance at CNP *G*_*CNP*_ versus 100/T. A band gap of *E*_*g*_ = 36.6 ± 3 meV is obtained for *V*_*ig*_ = −1 V. Solid line is the fitting curve based on the thermal-activation formula.

The freezing point of DEME-TFSI ionic liquid is at about *T* = 180 K, and precaution is needed while cooling down the device near the freezing point to avoid possible damage due to the volume change of ionic liquid below the freezing point. In addition, since the ions are frozen below 180 K, one would need to warm up the ionic liquid to temperature higher than the freezing point to change the charge doping level. Figure 2(a) plots the sheet resistance *R*_□_ versus *V*_*bg*_ at three different ionic liquid side gate voltages *V*_*ig*_ values. The device was first warmed up to *T* = 220 K followed by setting a new *V*_*ig*_ value. Due to the slow motion of ions, we typically waited for at least 30 minutes till the ions reached steady state, and then it was slowly cooled down to *T* = 20 K for resistivity and TEP measurements. The progressive shift of the charge neutral point (CNP) to higher *V*_*bg*_ values as *V*_*ig*_ goes from +1.5 V to −1.5 V is an indication of effective charge doping in the BLG. Both the peak location and also *R*_□_ at CNP shows nearly linear dependence on the *V*_*ig*_ as demonstrated in Fig. 2(b). The linear fit to the data points give *dV*_*bg*_/*dV*_*ig*_ = −47.5 at *T* = 300 K, which infers that the ionic liquid gating effectively introduces 47.5 times more charge carriers than regular bottom gate *V*_*bg*_ via SiO_2_ (300 nm)/Si if the same voltage bias is used. At *T* = 20 K, *dV*_*bg*_/*dV*_*ig*_ reduces to −31, which suggests that nearly 30% of induced charges at the surface become immobile at lower *T*. We also notice that the full-width half maximum (FWHM) of the *R*_□_ − *V*_*bg*_ curve is getting smaller as the *V*_*ig*_ goes more negative in magnitude. This may imply a nontrivial charge doping effect via ionic liquid gating, where the charge puddle effects can be an important issue. From the *T*-dependence of conductance at CNP *G*_*CNP*_ (open square in Fig. 2(c)), a band gap of *E*_*g*_ about 36.6 ± 3 meV at *V*_*ig*_ = −1 V and *V*_*bg*_ = +23 V is obtained by fitting to the thermal activation formula *G*_*CNP*_ = A exp[−*E*_*g*_/*k*_*B*_*T*] + C, where A and C are *T* independent constants. The resulting fitting curve is shown as solid line in Fig. 2(c).

**Figure 3:**
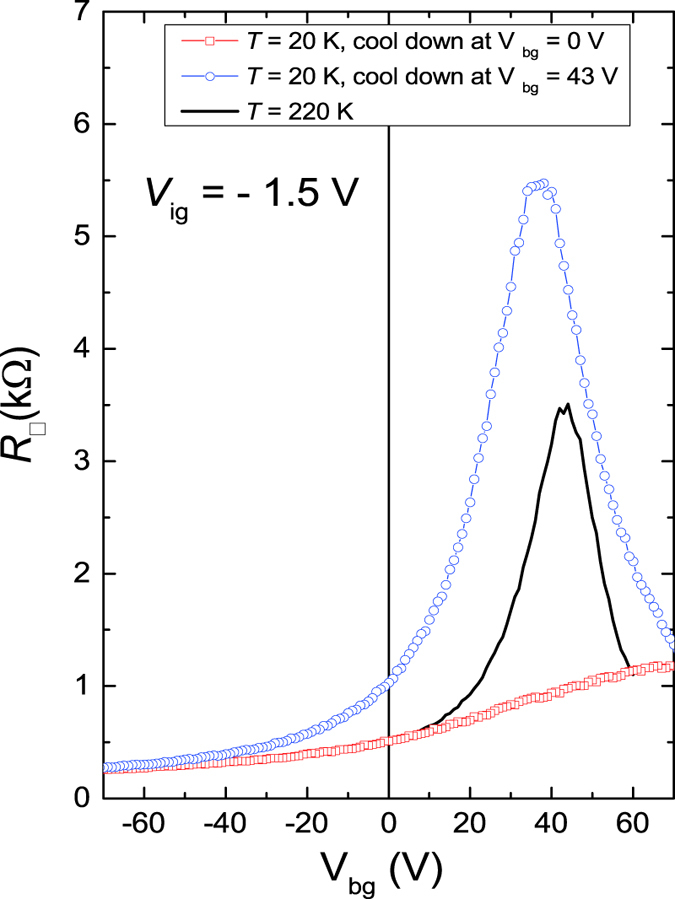
Sheet resistance *R□* versus bottom gate *V*_*bg*_ at *T* = 20 K and *V*_*ig*_ = −1.5 V. Blue and red data points are the data taken at *T* = 20 K after cooling down the device at *V*_*bg*_ = 0 V and +43 V, respectively. The solid black line is the *R*_□_ − *V*_*bg*_ curve at *T* = 220 K, which is above the freezing point of the ionic liquid DEME-TFSI.

In order to further look into the issue about FWHM in the *R*_□_ − *V*_*bg*_ curve, we carried out a series of experiments by cooling down the device at different *V*_*bg*_ values after setting a new *V*_*ig*_ value. Surprisingly, we found that the *R*_□_ − *V*_*bg*_ curve at low *T* is strongly dependent on the initial state before cooling. When the Fermi level was kept at CNP during the device cool-down, the *R*_□_ − *V*_*bg*_ curve shows much larger *R*_□_ at CNP and smaller FWHM compared to the one with Fermi level away from the CNP during the cool-down. This is demonstrated in Fig. 3, where all the three curves are measured at *V*_*ig*_ = −1.5 V. The black curve is the data at *T* = 220 K above the DEME-TFSI freezing point, which indicates that Fermi level can be shifted to the CNP by applying a *V*_*bg*_ = +43 V. The blue and red curves are the *R*_□_ − *V*_*bg*_ data at *T* = 20 K with application of *V*_*bg*_ = +43 V and 0 V during the cool-down, respectively. Remarkably, the blue curve (open circle symbol) shows a FWHM of about 30 V and a large peak at *V*_*bg*_ = +38 V while the red curve (open square symbol) only shows a gradual increase in *R*_□_ with increasing *V*_*bg*_ up to +70 V without attaining a local maximum. This finding suggests that the charge puddles condition in BLG is strongly influenced by the ionic liquid, which requires careful control over the cooling procedure. One likely explanation is due to the non-uniform charge distribution in the EDL on the surface of BLG, which is likely due to the defected condition of the interface between the BLG and ionic liquid. By setting the Fermi level at CNP during the cool-down, it minimizes the charge screening effect with the mobile ions and thus introduces smaller charge puddles at low T resulting in a better device performance.

**Figure 4:**
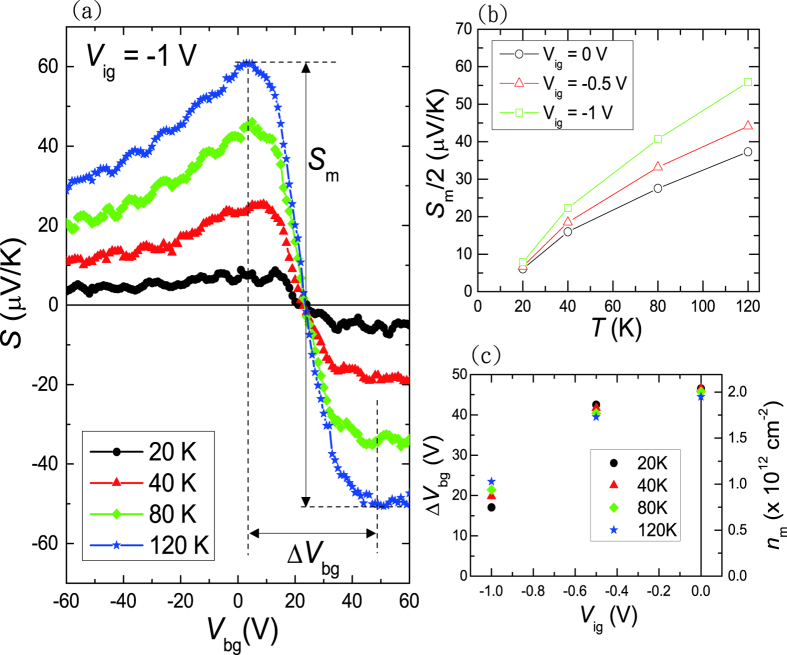
(**a**) TEP *S* versus *V*_*bg*_ at *V*_*ig*_ = −1 V at 4 different temperatures. (**b**) The temperature dependence of *S*_*m*_/2 at three different side gate voltages of *V*_*ig*_ = 0, −0.5 and −1 V. (**c**) Δ*V*_*bg*_ and the corresponding *n*_*m*_ as a function of *V*_*ig*_. A progressive decrease in Δ*V*_*bg*_ with increasing magnitude of *V*_*ig*_ is shown.

By using the same cool-down sequence of setting Fermi level at CNP, we also performed TEP measurement on a BLG device with ionic liquid gating. The local heater current was modulated at a low frequency *ω*, and the TEP signals were taken by a lock-in amplifier at second harmonic 2*ω*. The temperature gradient across the BLG was determined by two local thermometers made of thin gold nanowires. The resulting TEP *S* versus *V*_*bg*_ at *V*_*ig*_ = −1 V and 4 different temperatures is shown in Fig. 4(a), where the definitions for the separation of the local extremes Δ*V*_*bg*_ and TEP *S*_*m*_ are given. Both the *S* − *V*_*bg*_ and the *R*_□_ − *V*_*bg*_ curves at lower temperatures show nearly no hysteresis when sweeping *V*_*bg*_. When Fermi level is tuned to the CNP, the TEP is nearly zero. The TEP reaches local extreme values as the Fermi level shifts away from the CNP. We note that all the *S* − *V*_*bg*_ curves shows only single sudden drop in *S* = 0 at ~*V*_*bg*_ = +23 V suggesting that most of the thermoelectric signals come from the ionic liquid gating effective region. The contribution from non-effective regions outside of the opening window shown in Fig. 1(d) with CNP at 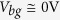 is thus negligible. On the other hand, the Δ*V*_*bg*_ is about 40 V consistent with the FWHM of the *R*_□_ − *V*_*bg*_ curve shown in Fig. 3. The maximum TEP *S*_*m*_/2 grows with increasing temperature as shown in Fig. 4(b), where *S*_*m*_/2 − T curves at *V*_*ig*_ = 0, −0.5, and −1 V are plotted. A clear enhancement of the TEP is observed when applying ionic liquid side gate *V*_*ig*_. At *T* = 120 K, the TEP *S*_*m*_/2 increases by about 40% from 30 *μ*V/K at *V*_*ig*_ = 0 to 42 *μ*V/K at *V*_*ig*_ = −1 V.

The enhancement of TEP in BLG device using ionic liquid does not give a larger TEP compared to the dual-gated BLG device using hBN as top gate dielectric^9^. One important factor is the electron-hole puddles effect, which turns out to be more significant in ionic liquid gating device as pointed out earlier. This can be seen more clearly from the variation of Δ*V*_*bg*_ at different *V*_*ig*_ values shown in Fig. 4(c). Δ*V*_*bg*_ equals about 45 V at *V*_*ig*_ = 0 and systematically drops to 20 V at *V*_*ig*_ = −1 V, which remains bigger than other dual-gated BLG devices typically showing Δ*V*_*bg*_ ≤ 10 *V*^9^,^16^. It is known that the electrons and holes both contribute to the total conductivity, but they would cause a compensation effect to the TEP signal^17^,^18^,^19^,^20^. By taking a simplified two-band model with both electron and hole pockets at the Fermi level^9^, the total TEP can be expressed as 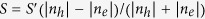, where *S*′ and *n*_*e*(*h*)_ are the TEP for each band and the electron (hole) density, respectively. When the Fermi level is at the CNP, the total *S* equals zero. TEP *S* starts to increase rapidly as the Fermi level shifts away from the CNP, and it reaches a local extreme when one of the pockets was fully depleted. Therefore, the density *n*_*m*_ at the local extremes in *S* − *V*_*bg*_ curve is a good indication of the amount of charge puddles near the CNP in a BLG device. *n*_*m*_ can be estimated using the formula of *n* = *C*_*g*_/*e V*_*bg*_, where *C*_*g*_ is the gate capacitance and 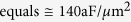 determined from the Hall effect measurements. As shown in Fig. 4(c), *n*_*m*_ equals 1.0 × 10^12^ cm^−2^ at *V*_*ig*_ = −1 V, which is more than two-fold larger than that in a dual-gated BLG device using hBN as top gate dielectric. As suggested by the theoretical calculations^9^,^21^, the magnitude of TEP enhancement is largely dependent on *n*_*m*_, where significant TEP enhancement can be achieved in devices with much lower *n*_*m*_ values. The charge puddles effect near CNP appears to be less significant at higher *V*_*ig*_ values, which is likely associated with the improved charge distribution in the EDL on the surface of BLG under finite electric bias.

In summary, we have demonstrated the feasibility of using ionic liquid as a top gate to induce a band gap in a BLG. We found that the cooling procedure of the device can strongly affect the amount of charge puddles in a BLG possibly associated with the non-uniform charge distribution in the EDL of ionic liquid. A lowest level of charge puddles can be achieved by cooling down the device while keeping the Fermi level at the CNP. In this way, we successfully demonstrate the enhancement of TEP by nearly 40% with a merely −1 V application to the ionic liquid side gate. For further enhancement in TEP, solution to the reduction of charge puddles near CNP and a cleaner graphene surface condition with proper device cooling procedure are both needed and require more experimental efforts.

## Additional Information

**How to cite this article**: Chien, Y.-Y. *et al.* Thermoelectric Power in Bilayer Graphene Device with Ionic Liquid Gating. *Sci. Rep.* **6**, 20402; doi: 10.1038/srep20402 (2016).
